# Cytomegalovirus colitis presented with triserial longitudinal hemorrhage in an autopsy case of adult T‐cell leukemia

**DOI:** 10.1002/ccr3.2423

**Published:** 2019-09-18

**Authors:** Kenji Yorita, Akira Ishihara, Takanori Toyama

**Affiliations:** ^1^ Department of Diagnostic Pathology Japanese Red Cross Kochi Hospital Kochi‐shi Japan; ^2^ Department of Anatomic Pathology Miyazaki Prefectural Nobeoka Hospital Miyazaki Japan; ^3^ Department of Internal Medicine Miyazaki Prefectural Nobeoka Hospital Miyazaki Japan

**Keywords:** adult T‐cell leukemia, colitis, cytomegalovirus, longitudinal ulcer

## Abstract

Longitudinal mucosal and submucosal hemorrhage along the taeniae coli is a potential colonic manifestation of cytomegalovirus infection in immunocompromised patients. When diagnosing cytomegalovirus colitis in immunocompromised patients, endoscopic biopsy on taeniae coli seems effective for viral detection.

## Graphical abstract

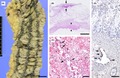

## 1 WHAT INFECTION WAS PRESENT IN THE COLON?

**Figure 1:**
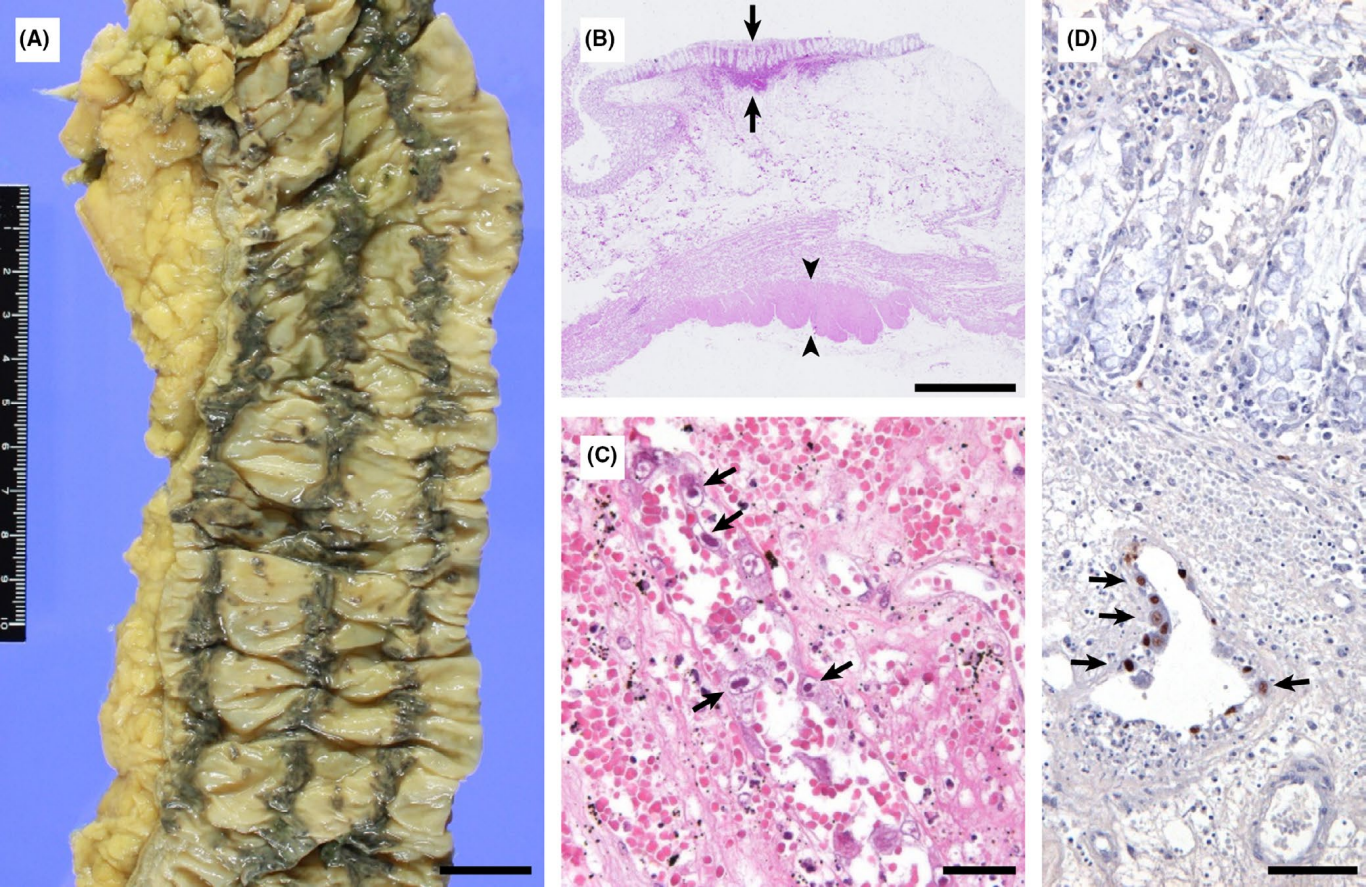
Three longitudinal lines are observed throughout the colon (A) in an autopsy case with adult T‐cell leukemia. The colonic lines show mucosal and submucosal hemorrhage [arrows, (B)] present along the taeniae coli [arrowheads, (B)]. The hemorrhagic areas include marked CMV endothelialitis, showing endothelial nuclei with inclusion bodies [arrows, (C)] positive for anti‐CMV antibody [arrows, (D)]. Scale bar in A, B, C, and D is 2 cm, 2 mm, 50 μm, and 100 μm, respectively

A 72‐year‐old woman had acute cholecystitis 1 month ago, and abnormal cells in the peripheral blood yielded a diagnosis of acute‐type adult T‐cell leukemia (ATL). Antibiotics and zoledronic acid were administered. Since her leukocyte count increased to 5.0 × 10^10^/L; chemotherapy was initiated but was ineffective. Melena or abdominal pain was absent. Autopsy revealed ATL involving various organs and a disseminated cytomegalovirus (CMV) infection. She died of the tumor. Throughout the colon, three longitudinal lines (Figure 1A) showing mucosal and submucosal hemorrhage (arrows, Figure 1B) were present along the taeniae coli (arrowheads, Figure 1B). The hemorrhagic areas included marked CMV endothelialitis (Figure 1C,D); however, the CMV infection was markedly lesser in the nonhemorrhagic colonic areas.

The colon is a common site for CMV infections. The prominent endoscopic finding is subepithelial hemorrhage,^1^ and colonic triserial longitudinal hemorrhage has not been reported. This finding was clinically insignificant; however, it may help determine the mode of CMV infection in the colon. In our case, CMV might have infected endothelial cells in the colonic wall along the taeniae coli because it is prone to be ischemic,^2^ and colonic blood flow tended to decrease by hyperviscosity induced by leukemia cells.

## CONFLICT OF INTEREST

The authors state that they have no conflict of interest.

## AUTHOR CONTRIBUTION

KY: drafted the manuscript and contributed to autopsy and pathological diagnosis. AI: assisted in drafting the manuscript and pathological diagnosis. TT: diagnosed and treated the patient.
